# Cauda equina syndrome secondary to leptomeningeal metastases from recurrent primary peritoneal carcinoma

**DOI:** 10.3332/ecancer.2018.814

**Published:** 2018-02-26

**Authors:** Zhen Ni Zhou, Chelsea Canon, Cathleen Matrai, Eloise Chapman-Davis

**Affiliations:** 1 Department of Obstetrics and Gynecology, Weill Cornell Medical College––New York Presbyterian Hospital, New York, NY 10065, USA; 2 Department of Pathology, Weill Cornell Medical College––New York Presbyterian Hospital, New York, NY 10065, USA; 3 Department of Obstetrics and Gynecology, Division of Gynecologic Oncology, Weill Cornell Medical College––New York Presbyterian Hospital, New York, NY 10065, USA

**Keywords:** cauda equina syndrome, primary peritoneal carcinoma, leptomeningeal metastasis

## Abstract

The patient is a 42-year-old woman with metastatic primary peritoneal carcinoma and known brain metastases, who subsequently developed cauda equina syndrome after presenting with ataxia, lower extremity weakness, and bowel and bladder incontinence secondary to leptomeningeal metastasis after treatment with neoadjuvant chemotherapy, surgical debulking, and adjuvant chemotherapy. Metastases to the central nervous system (CNS) and leptomeninges are rare events in epithelial ovarian and primary peritoneal carcinomas as these tumours do not have a predilection for the CNS. Cauda equina syndrome is often characterised by gait disturbances, bowel and bladder dysfunction, saddle anaesthesia, and lower extremity muscle weakness. In patients with known metastatic gynaecologic carcinomas presenting with nonspecific neurologic symptoms, cauda equina syndrome should remain high in the differential diagnosis.

## Introduction

Primary peritoneal carcinomas, accounting for approximately 7–13.8% of ovarian carcinomas and histologically identical to advanced epithelial ovarian cancer (EOC), are characterised by diffuse peritoneal disease in the absence of an obvious primary site and either grossly normal ovaries or ovaries with minimal disease burden [1]. While metastasis to the central nervous system (CNS) secondary to malignant gynaecologic neoplasms is rare, leptomeningeal metastasis (LM) is even more uncommon and often detected late in the disease course. Despite recent advances made in the diagnosis and treatment of brain metastases, patient prognosis and survival remain poor. In this report, we describe an unusual case of a patient with metastatic primary peritoneal carcinoma and known brain metastases, who subsequently developed cauda equina syndrome after presenting with ataxia, lower extremity weakness, and bowel and bladder incontinence secondary to LM after treatment with neoadjuvant chemotherapy, surgical debulking, and adjuvant chemotherapy.

## Case

A 42-year-old woman with a longstanding history of tobacco use underwent an evaluation for persistent cough and shortness of breath. The patient was found to have recurrent pleural effusions requiring multiple thoracenteses, right-sided video-assisted thorascopic surgeries (VATS), partial decortications, and pleurodeses in the interim for symptom relief. An extensive workup was performed and initial immunohistochemistry (IHC) stains of tissue biopsy samples obtained from the VATS supported a diagnosis of poorly differentiated metastatic breast carcinoma.

**Figure 1.**
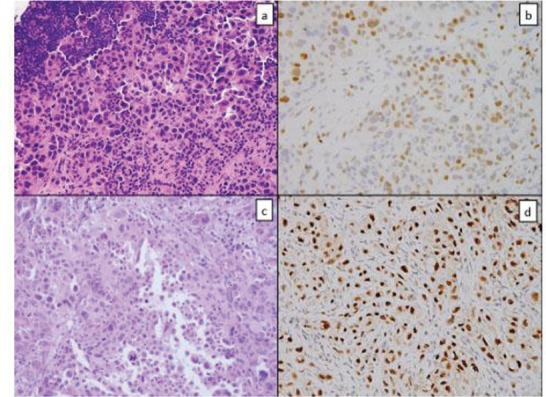
Tissue from a frozen section control of a mediastinal lymph node showed sheets of poorly differentiated carcinoma consisting of malignant cells with marked pleomorphism and a moderate amount of cytoplasm (a); GATA-3 immunostain showed patchy strong positivity (b); similar tumour cells were seen in the pericardial specimen (c); Pax-8 stain was strongly positive (d).

**Figure 2.**
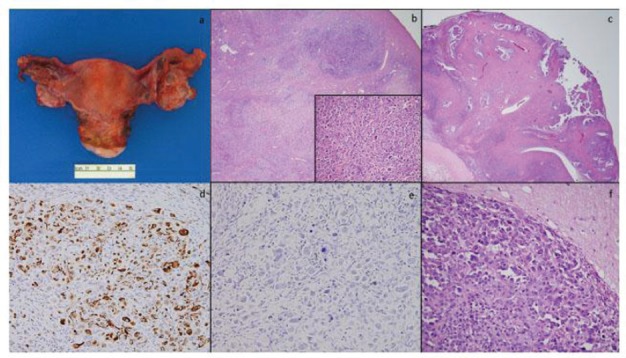
Total abdominal hysterectomy and bilateral salpingo-oophorectomy specimen showed a slightly enlarged left ovary with few cysts and was otherwise unremarkable (a); low-power microscopy (4×) of the left ovary showed tumour within ovarian parenchyma (b) and high-power (40×) magnification shows similar cells to those seen in the previous specimens (b, inset); the right ovary also showed areas of tumour with a classic papillary appearance (c); a WT-1 stain was diffusely positive (d); a BRST-2 stain was negative (e); brain metastases showed highly pleomorphic cells with features of serous carcinoma extending into adjacent glial tissue (f).

**Figure 3.**
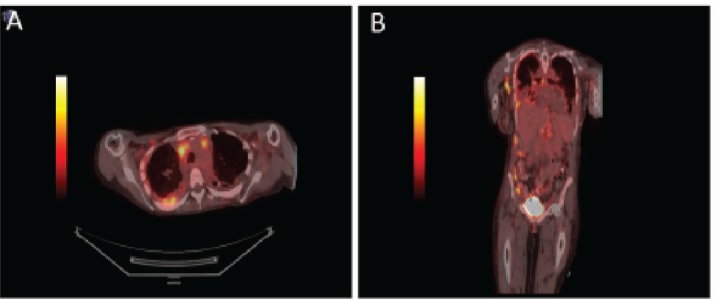
PET/CT body showing diffuse enhancement of disease. Axial views of the body on PET/CT demonstrating (a) intrathoracic lymphadenopathy and FDG avid right pleural thickening. Coronal view of demonstrating axillary lymphadenopathy, intrathoracic lymphadenopathy, right pleural thickening, FDG-avid caking on ascending colon and right lateral abdominal wall implant (b).

Prior to initiating treatment for presumed breast cancer, the patient presented to the emergency department (ED) with acute respiratory distress and tachycardia. She underwent the placement of a pericardical window for a moderate pericardial effusion seen on echocardiogram as well as drainage of a loculated right-sided pleural effusion seen on chest computed tomography (CT), respectively. Interestingly, the IHC staining profile of the fluid revealed tumour cells with classic findings for an ovarian serous carcinoma, which were later confirmed in the patient’s resection specimen and not consistent with a primary breast carcinoma (Figures 1 and 2). The patient underwent further imaging with a positron emission tomography (PET)/CT scan that revealed extensive lymphadenopathy, diffuse peritoneal implants, and omental caking in the absence of obvious evidence of adnexal or uterine disease (Figure 3). Based on these findings, in conjunction with an elevated serum cancer antigen-125 (CA-125) level (11,048 U/mL), the diagnosis of stage IVB primary peritoneal cancer was ultimately made almost 10 months after her initial evaluation for shortness of breath.

The patient was given six cycles of neoadjuvant chemotherapy with carboplatin (area under the curve 6) and paclitaxel (175 mg/m^2^). The serum CA-125 level decreased to 625.7 U/mL, along with the improvement of clinical status and regression of tumour burden. The patient then underwent an interval debulking surgery consisting of a robotic assisted total hysterectomy, bilateral salpingo-oophorectomy, and omentectomy that resulted in R0 resection. Following the surgery, she received additional four cycles of carboplatin and paclitaxel with normalisation of the CA-125 level (23.2 U/mL). She desired a treatment break due to extensive side effects stemming from chemotherapy despite evidence of small volume disease on imaging. At this point, the patient underwent genetic testing and was found to have a *p53* mutation and a *BRCA2* variant of unknown significance.

**Figure 4.**
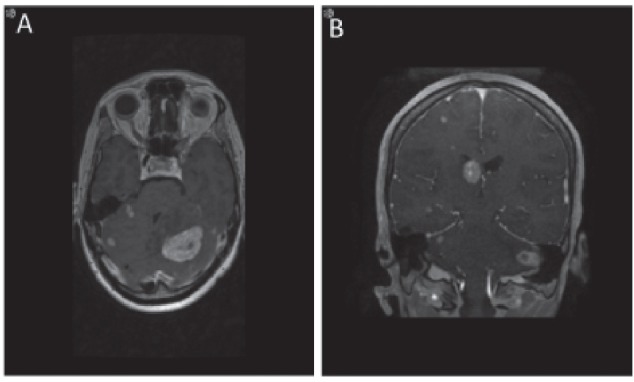
MRI brain demonstrating metastases to brain. Axial (a) and coronal (b) views of the brain demonstrating the presence of brain metastasis.

**Figure 5.**
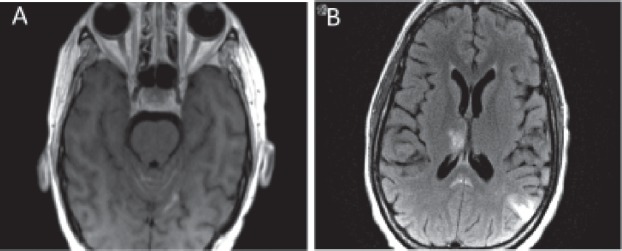
MRI brain showing leptomeningeal disease. (a) T1 axial MRI with contrast depicting new enhancement along superior cerebellar folia compatible with leptomeningeal disease. (b) Abnormal leptomeningeal enhancement along posterior margin of splenium of corpus callosum.

The patient desired expectant management, but the serum CA-125 level was found to be elevated at 243.5 U/mL after two months. Thus, she was restarted on chemotherapy with doxorubicin (40 mg/m^2^) for platinum-resistant disease. Two days after completing the first cycle, she presented to the ED after having a seizure at home. New brain metastases near the hypothalamus with surrounding vasogenic oedema were detected on magnetic resonance imaging (MRI) scans of the brain (Figures 4 and 5). The patient subsequently underwent left suboccipital craniotomy and tumour resection followed by whole brain radiation therapy (WBRT). IHC performed on the specimen was consistent with metastatic adenocarcinoma consistent with the origin from the known primary peritoneal carcinoma. A multidisciplinary meeting was held and the decision was made to proceed with palliative chemotherapy with single-agent gemcitabine (800 mg/m^2^).

After completing the third cycle of gemcitabine, the patient presented to the ED complaining of acute onset gait instability, bilateral leg numbness and weakness, and bowel and bladder incontinence. She was diagnosed with cauda equina syndrome after an MRI of the spine was notable for leptomeningeal disease in the conus medullaris; she was given dexamethasone for acute treatment. Lumbar puncture was not performed given obvious disease involvement on imaging studies. The patient also received palliative radiation in addition to medical management for CNS symptoms and neuropathic pain. Unfortunately, the patient never had resolution of her neurologic symptoms and died two months later.

## Discussion

Primary peritoneal carcinomas develop* de novo* from the abdominal and pelvic and account for up to 15% of EOCs. While some cases have been shown to arise from independent malignant transformation at multiple peritoneal sites simultaneously, recent studies suggest that up to 50% of these cases arise in the fallopian tube fimbriae [2]. Clinically and histologically, primary peritoneal carcinomas are indistinguishable from EOCs. The delay in diagnosis was complicated by the unusual presentation, conflicting IHC profile results, and the patient’s initial non-compliance with recommendations for follow-up.

The patient was initially diagnosed with metastatic breast cancer. However, subsequent pathology revealed patchy strong positivity for *GATA-3* and diffuse positivity for *BRST-1*. While *GATA-3*, which has high specificity in breast and urothelial carcinomas, is rarely positive in primary ovarian carcinomas, up to 6% of ovarian serous carcinomas may express *GATA-3* [3]. *BRST-1* expression, which was positive in the original specimen, is less specific for serous ovarian carcinomas than *BRST-2* expression [4]. Pax-8 positivity has not yet been demonstrated in primary breast carcinoma and studies have shown a positivity rate of 0% in these tumours [5]. The presence of both *PAX-8* and *WT-1* and the absence of BRST-2 on secondary specimens helped to confirm the diagnosis as primary Mullerian origin.

Involvement of the CNS is uncommon in EOCs with an incidence ranging from 0.29% to 4.5% [6, 7]. Often, metastasis to the CNS is detected late in the disease process and despite advances in treatment options and radiation therapies, patient survival and prognosis remain poor [7]. Although studies have shown that patients with recurrent platinum-sensitive disease can benefit from retreatment with a platinum agent, alternative agents and non-platinum based chemotherapy are potential therapeutic approaches in patients with primary refractory or recurrent platinum-resistant primary peritoneal cancers and EOCs.

Our patient presented with seizures and was found to have brain metastases in the setting of receiving second-line treatment. Treatment subsequently shifted to WBRT for palliation. Brain metastases are a rare occurrence in patients with gynaecologic malignancies and are associated with poor prognosis. In a recent retrospective review of patients with brain metastases and gynaecological cancers, treatment with multimodal therapy including surgical resection, WBRT, and chemotherapy was associated with improved survival, particularly in the setting of solitary brain lesions and controlled extracranial disease [8]. Due to the young age of our patient and presumed low volume extracranial disease, aggressive treatment was pursued with WBRT and surgical resection. Options for subsequent chemotherapy in patients with brain metastases in the setting of primary gynaecologic malignancy are limited. The blood–brain barrier (BBB) integrity is thought to limit delivery of large hydrophilic agents to brain metastases, therefore presenting an obstacle in choosing agents to use. Gemcitabine was selected as a third-line treatment due to presumed platinum resistance in this patient as well as penetrance through the BBB [9]. The efficacy of gemcitabine as single-agent or combination therapy has been evaluated in several clinical trials, and tumour remission has been observed in patients with sensitivity and resistance to both platinum and paclitaxel [8–10]. Gemcitabine has also been used to treat patients with brain metastases and the response of brain metastases to chemotherapy generally has been similar to that of tumours of similar histology located extracranially [10].

Unfortunately, after only three cycles of third-line treatment, our patient presented with symptoms of cauda equina syndrome, i.e., bilateral lower extremity numbness and weakness, gait instability, and bowel and bladder incontinence, leading to the diagnosis of leptomeningeal disease. These symptoms can be explained by infiltration and compression of the conus medullaris secondary to metastatic disease. LM most commonly arises from breast and lung cancers as well as melanomas. However, LM is an incredibly rare sequela of primary peritoneal carcinoma. Patients with LM classically present with multifocal neurologic symptoms and signs, and the diagnosis is made via MRI, cerebrospinal fluid cytologic analysis, or both. As reports of LM secondary to EOCs are rare, treatment strategies are based on published case reports. Treatment is palliative in nature and can involve focal radiation to symptomatic sites, and systemic chemotherapy with agent(s) that cross the BBB.

Intrathecal chemotherapy with methotrexate has been used successfully to treat LM from ovarian cancer [6]. However, its effectiveness may be limited and its superiority over systemic treatment has not been established in randomised trials. In addition, no standardised method of measuring response to therapy or evaluating disease progression of LM has been well established, both of which are essential for validating the continued use of aggressive treatments such as intrathecal chemotherapy, which is associated with meningitis and myelosuppression [11, 12].

## Conclusions

In summary, we have presented a case of cauda equina syndrome secondary to infiltration of the conus medullaris by leptomeningeal disease in a patient with stage IVB primary peritoneal carcinoma and brain metastases. Although rare, the possibility of LM from primary peritoneal carcinoma exists. At present, there is no standard of care with regard to treatment of recurrent platinum-resistant primary peritoneal carcinoma or leptomeningeal disease. Treatment using alternative agents such as gemcitabine, palliative irradiation, and intrathecal chemotherapy should be considered if the patient is otherwise in good condition. Due to the rarity of brain metastases in patients with gynaecologic malignancies, this case highlights the importance of utilising an integrated, multidisciplinary team based approach in order to provide the best possible patient care.

## Conflicts of interest

The authors have no conflicts of interest to report.
